# Static Body Balance in Children and Expert Adults Ballroom Dancers: Insights from Spectral Analysis of Shifts

**DOI:** 10.3390/biology10121291

**Published:** 2021-12-08

**Authors:** Antonio Cicchella

**Affiliations:** 1 Department for Quality-of-Life Studies, University of Bologna, 40126 Bologna, Italy; antonio.cicchella@unibo.it; 2 International College of Football, Shanghai Tongji University, Shanghai 200092, China

**Keywords:** development, children, balance, motor control, spectral analysis

## Abstract

**Simple Summary:**

The development of balance in children engaged in a particular and challenging task such as dance, is influenced by several factor, including growth of body and limbs, development of the central nervous system, and training. Comparing a group of experienced dancers and child dancers, we found that gender differences are overshadowed by technical training in adults. The dominance of the vestibular system (centralized and efficient fine control system) is apparent in adults from the static equilibrium tests carried out in this study. In children, however, a greater use of the somatic-sensory system (stereotyped and superficial) was recorded. A recommendation for practice, therefore, can be to train children in balance exercises with their eyes open, while adults could train with their eyes closed, to stimulate the use of the relevant balance subsystem. A limitation of our study may be, as is the case for most of the previous research on the topic, that we measure balance in lab conditions and not in real life conditions.

**Abstract:**

Objective: The purpose of this study was to assess the differences in maintaining body balance (influence of different sensorial sub-systems) in a representative sample of active Dance Sport competitors (children and adults). Methods: Overall, 13 children and 15 high-level adults sport dancers underwent a static equilibrium test on a force platform, in which 17 different parameters were examined, including a spectral analysis of shifts using an FFT algorithm that can assess the contribution of different somatic-sensory systems on maintaining body balance. Results: Younger subjects rely on their somatic-sensory reactions to maintain their balance, while adults rely more on the vestibular system, according to shifts’ spectral analysis. No differences were noted between the male and female participants. Conclusions: Children predominantly use the somatic-sensory system in body balance, while adults make more use of the vestibular system. According to these results and due to the trainability phases, exercises that challenge the somato-sensorial system are recommended to train balance in young dancers, while exercises that challenge the vestibular system are recommended in adult dancers which who have not developed exceptional somato-sensory balance abilities during their growth and training history.

## 1. Introduction

Body balance is influenced by a cohort of physiological systems and dance has been shown to be able to alter brain neuroplasticity to improve body balance [1]. Visual [2], vestibular (inner ear) and proprioceptive (afferents from muscle spindles and inner joints sensors) systems develop with aging, especially from the range of 4 to 12 years old (with a peak growth after the 7 to 8th year). It has been observed that young females tend to outperform young males across all balance tests in this age range [3]. Information from the vestibular, visual and proprioceptive systems afferents are integrated into the brain stem, specifically into the cerebellum [4]. This finding was also recently confirmed in studies involving children with special diseases [4]. Dancers include balance exercises in their daily routines, for example when performing on pointe techniques in classical ballet for choreography. In ballroom dance, balance exercises are included in children dancers’ training, for the purpose of improving posture and the speed of performance [5].

It is widely accepted that the coordinate capacity of balance is fundamental for performance and has been studied in most sports [6]. Less information is available in literature about the balance capacities of children dancers [7], including in literature regarding ballroom dance [8]. Thus, is important to investigate the development of balance, comparing two extreme (for capabilities) groups: children and expert adults. This distinction will allow for the better identification of the relevant parameters involved in balance control. Furthermore, available studies are limited to the assessment of body balance through sway analysis and usually consider only linear measures. In Dance Sport (ballroom dancing), balance capability becomes even more important compared with other dance techniques, because dancers must constantly balance when performing in pairs, and balance is indeed an evaluation factor for the competition’s judges.

Postural equilibrium related to age and gender has also been widely investigated. Regarding gender differences, some studies indicated that females had better balance capacities than their male counterparts, with this being especially true up until the developmental age (e.g., age of menarche) [3,9,10], the point at which differentiation occurs due to an increase in strength in males [11,12,13,14]. From the age of menarche onward, better static balance was demonstrated in males compared to females, because balance also depends on strength capabilities. Higher lower-limb strength in males can explain the sex-differences found in non-athletic or specially trained populations. These results were investigated in a large study involving 2024 males and 1892 females with age spanning 4–74 years old, with no differences in static balance between males and females from 4 to 39 years of age [15].

Body balance is dependent on the development of the biological structure of an individual. The cerebellum is the integrative brain stem structure involved in the maintenance of body balance.

Total cerebellum volume follows a developmental trajectory, peaking at 11.8 years of age in females and 15.6 years in males [16].

Thus, it is clear that cerebellar development is another factor that contributes to the control of equilibrium with aging, until maturity [12,14]. An important observation that appear in the literature is that younger subjects make greater use of stereotyped automatic strategies, which rely most on somato-sensory system (vision and joint’s proprioceptors) [17,18].

Young children are unable to systematically suppress the influence of inputs derived from conflicting proprioceptive inputs or vision [18] (e.g., moving or unstable ground and moving optical landscape). However, there is a lack of consensus in the literature regarding the dependency of dancers on vision for balance control. In ballet dancers, vision has been found to be the primary system for balance control compared to non-dancers [19]. This result is not in accordance with another study, which found that dancers were significantly less dependent on vision but used more proprioception than untrained subjects [20]. According to this latter study, professional dance training appears to shift sensorimotor dominance from vision to proprioception, and this shift seems more marked in males than in females [20].

The importance of visual information in the process of dancer´s postural control was also observed in another study, which found increased sway without visual feedback. The same study measured sway’s entropy, observing more irregular characteristics of postural sway in ballet dancers representing more automated postural control [21]. However, not all studies agree that dancers have better balance than non-dancing adults [22].

Contradictory results were found regarding the relationship between anthropometric factors and static equilibrium, especially in studies that consider gender differences in static balance. Some authors [11] found that, in females, height and better balance is inversely correlated, (e.g., higher the subject, worsen the balance) while others [23] asserted that no differences exist between male and females, with height representing a worsening factor for balance in both genders. Thus, the anthropometric parameter that most closely correlates with equilibrium seems to be height [11,24]. In fact, it is methodologically recommended that a normalizing procedure should be used for height to neutralize balance variables among subjects [24]. The limitation of existing studies on the relationship between static equilibrium and dance indicates that training in this discipline can attenuate gender differences [25], while differences in age brackets remain unvaried [26]. It is, however, recognized that professional dancers achieve better results in the various postural tests, as compared with non-dancer subjects [27,28,29,30]. Some authors [21,31] hypothesize that dance training shifts the control of balance towards vestibular dominance. Therefore, dance lends itself well as a paradigm to emphasize, and thus study, the evolution of balance during growth.

A few studies thus far have been carried out on the population of children sport Ballroom Dancers [31]. In fact, there are no known studies that make a comparison between postural equilibrium in Dance Sport competitors of different ages. This also leaves unexplored the relationships between anthropometric growth in the subjects and of the development of static equilibrium. A comparison study between expert adults and children- novices in ballroom dancers has not been performed, so it is not yet understood how the different balance-maintenance systems work in this kind of dance. Dance Sport is very challenging for the balance, due to the rapid head rotations, high-heels usage, and fast movements, and thus presents an appropriate setting to study the development of body-balance capacities.

Our aim was to study the differences between children and adult ballroom dancers, and to investigate the influence of the various sensorial sub-systems involved in the development of equilibrium between subjects of different ages and qualification by means of a shift spectral analysis. The hypothesis is that balance control in children is governed mainly through mechanisms based on somatosensory information, while in adults balance is governed by mechanisms of vestibular information. Spectral analysis can provide insight regarding the governing mechanism of balance in children and in expert adults.

## 2. Materials and Methods

### 2.1. Participants

Participants belonged to two different age groups, including a group of 13 children, age 9.8 ± 1.7 years, height 139.9 ±13.5 cm (min. 133.15; max. 146.65 cm), weight 34.8 ± 8.5 kg, BMI 17.6 ± 3.4 and 15 adults, age 24.6 ± 4.4 years, height 171.6 ± 7 cm (min 164.6; max 178.6 cm), weight 61 ± 8.7 kg, BMI 20.6 ± 1.6). Subject data are reported in Table 1.

Children were recruited in ballroom dance schools of the city of Bologna by contacting the teachers, while adults were recruited in a professional ballroom dance school by contacting the team manager. The recruited adults were high-level dancers, included in the top 100 WDSF (World Dance Sport Federation) Ranking List, with an average of 16 years of dance experience. The children had a competition ranking which ranged from basic level (Class C and B) to advanced level (Class A). Children trained at least 4 days a week, competed in events on weekends and had a minimum of 4 years of dance school experience. Professional dancers had been trained every day for at least 2 h. All participants read and signed a consent form before taking part in the study. For children, written consent was provided by both parents and the study was approved by the ethical committee of University of Bologna, which granted a written authorization to the study (NR. 9627, 23 January 2019).

### 2.2. Measures

Height was measured with a Harpenden anthropometer (Holtain, Crosswell, Wales, England) to the nearest 1 mm and body weight was measured with a Seca scale (model 700, Hamburg, Germany). To study the Center of Pressure (COP) sway and to extract the frequency parameters, a Kistler, 9286AA (Winterthur, Switzerland), force platform was used [32,33] with a sampling rate of 1000 Hz [34]. The “Sway” software, v.1.4.1. (BTS, Milan, Italy) was used for data acquisition and processing. Tests were performed barefoot as described previously [29,30]. Static balance is the most studied condition of balance in dancers. Static balance is related to dynamic balance and can be assessed with greater objectivity. A recent review selected 9 studies comparing dances and non-dancers at different levels of development, and found that 5 studies were performed in static, 2 in dynamic, and 2 in static and dynamic conditions [19].

Male and female participants were tested in a random order.

### 2.3. Procedures

Anthropometric parameters were measured. The subjects then underwent a static equilibrium test in the laboratory, as described previously [31,34,35]. Shifts, lengths, speeds of shifts and areas have been suggested as better indexes of balance [33]. In addition, we chose to include the oscillation frequency parameters because it is more sensitive to vestibular changes [35]. The test was illustrated to the participants ahead of time and they were allowed to practice 3 times on each leg before testing. The test consisted of one static equilibrium test in single-limb standing, carried out on one lower limb and then on the other one, randomly selected. The test lasted 30 s [36], with the free leg bent at the knee below the subject and held by the ipsilateral arm (while the other arm remained straight on the side), while the subject focused on a red spot with a diameter of 2.5 cm placed on the wall at eye’s height, at a 2.5 m distance. If the subject fell or lost their grip on the free leg (causing loss of position), the test was repeated, until the subject succeeded in maintaining the position for the time requested.

The following time-dependent parameters were analyzed [24,35,36]: Shift Range M-L (mm): medium-lateral COP, Shift Range A-P (mm): Anterior-Posterior COP, Total movement length (mm): total length of the run of COP, Shift speed (m/s): mean speed of COP, equivalent area (mm^2^): total area of COP (Center of pressure). The shift parameters are largely employed in balance assessment, because they are representative of the capacity to maintain postural stability and can highlight the occurrence of worsening due to pathologies or changes due to training and development. Shifts depend on the integrity and function of the proprioceptive, vestibular and vision system in children [3,4]. The shift parameters are of particular interest in developing subjects, and has been studied in girls practicing dance in the developmental age [3,24]. A rectangular type FFT (Fast Fourier Transform) with a window of 1024 points was employed, using the software Sway [37,38] to break down COP oscillation into the following three wave spectrums: Spectrum Px = middle-lateral oscillation frequencies spectrum, Spectrum Py = anterior-posterior oscillation frequencies spectrum, Spectrum D = COP shift oscillation frequencies spectrum with respect to its centerline, i.e., the axis around it oscillates.

For each of these frequency spectrums, the following frequency-dependent parameters are returned: Power Peak (mm^2^), Frequency at Peak (Hz): Frequency at maximum power intensity, Mean Power, Average spectrum frequency.

The Tetrax classification of the sway spectrum, obtained by Fast Fourier Transform, is reported in Table 2, according to the balance mechanism involved [39]. Various degrees of vestibular, somato-sensory and central efficiency can be discriminated by these frequency bands. The contribution of each balance subsystem to frequency content of the oscillatory signal was experimentally checked in previous studies [37,39], reporting how each balance subsystem influences the frequency content. The F1 band relates to the visual subsystem [40]. Low-medium frequencies (F2–4) are related to the peripheral vestibular subsystem, and high-medium frequencies (F5–6) are related to the somatosensory subsystem [41,42].

### 2.4. Analysis

For each subject and each parameter, the mean and std were calculated as the mean of the combined right and left limbs. To eliminate body-height influence on balance, data were normalized to body height [43]. Means and standard deviations were calculated. Spearman correlations and 2 × 2 ANOVA (Fisher post hoc) for groups and gender differences were performed with SPSS, version 14 (Armonk, NY, USA According to the number of comparisons (between the 17 parameters considered), a Bonferroni correction resulted in *p* < 0.003. Spearman correlations were performed between shift parameters and subject´s body height. The significance level was set at *p* < 0.05.

The frequency-dependent parameters were calculated using FFT transformation [44,45,46,47]. The global spectrum of static oscillation of the human body (0.01–3 Hz) can be divided into different ranges of postural frequency bands. These studies show that, at different bands of frequency, there are different levels of 2 postural sub-systems activity (vestibular system, somatic-sensorial). Therefore, the frequency content of the time–oscillation signal provides information regarding the sub-systems used to keep the postural balance. In our study, we used the Tetrax frequency-band classification [37,39]. According to this classification, the Average to Low bands frequencies (0.1–0.5 Hz) show a corresponding dominance of the vestibular system, while Medium to High bands (0.5–1 Hz) show a dominance of the somatic-sensory system (peripheral control system, which is automatic and stereotyped, and consequently less precise).

## 3. Results

In Table 3 the significant correlations in the children group between height, balance and spectral parameters are presented. Any correlations found for adults were between height and all the measured variables (r < 0.200, *p* > 0.5).

In Table 4 the results of COP sway are presented, with the 17 selected parameters, subdivided into time-dependent and frequency-dependent categories (Spectrums Px, Py, D). Differently from other studies in non-dancer children [3], where the effect of gender on sway was observed, in our sample of dancers, any differences between males and females for all considered parameters were found to be an effect of dance training.

Shift range and Frequency to Max medio-lateral power showed statistically significant differences between children and adults. All other examined parameter did not show any significant differences (*p* > 0.05).

Higher oscillation and frequency values were found in the Children group, compared with the adult group. As observed in a previous study [48], as oscillation frequency increases, higher anterior-posterior and medio-lateral accelerations occur. The children group showed different results in the equilibrium tests, in accordance with previous studies carried out both on non-dancers [12,14,16] and on dancers [25].

## 4. Discussion

The Tetrax system can discriminate between vestibular, proprioceptive, and the visual contributions to balance by means of spectral analysis. It must be noted that in our study, the frequency of the maximum power in the Px spectrum, both for children and adults, falls in the low frequency band (0.01–0.1 Hz) of the Tetrax system [37,39]. The maximum power measure in this range corresponds to the postural oscillation spectrum of healthy subjects, standing erect and still. The average Frequency values of the Px spectrum for both groups are in the Medium–Low frequency band (0.10–0.50 Hz) of the Tetrax classification system [37,39] and correspond to the use of the vestibular system to maintain the equilibrium in a healthy body in a state of instability or fatigue.

Interestingly, while the average for the adults stood at the center of this range, (0.38 Hz), the children’s parameter (0.50 Hz) was located at the edge of the Medium–High frequency band (0.50–1.00 Hz). The results of our study suggest that adults rely more on the vestibular system, and children on the somato-sensory system, according to the Tetrax classification reported in Table 3.

Our hypothesis that balance control in children is governed mainly by mechanisms based on somatosensory information, while in adults balance control relies on mechanisms of vestibular information is confirmed by the results.

Another finding was that males and females do not show significant differences, and this finding is likely an effect of dance training. As the literature is controversial on this point, our study agrees with the hypothesis that there are no differences between males and females.

The development of physiological structures, which are involved in balance maintenance, peaks at 11.8 year for females and 15.6 years for males [18].

Furthermore, the development of balance capacities can be attributed to training. Our children’s groups are younger than this age range, while our expert adults group is much higher than this age range beyond. We are thus almost sure that, in the children group, developmental changes occur. Children demonstrated both higher oscillation amplitudes and frequencies in the Px spectrum, due to a less stable equilibrium. This means that the older the subjects, the smaller and lower the frequency of the oscillation, which translates into a better control of equilibrium [5,12,16]. These studies agrees with the hypothesis that aging improves the balance.

Therefore, younger subjects needed to mobilize their somatic-sensory reactions to keep their balance, using the motor apparatus to stabilize. These somato-sensory reactions include spinal reactions (reflexes) [49]. It can be presumed that these later reactions contribute to a vestibular system that is still under development. This hypothesis agrees with the reports of various authors [12,17,50]. Moreover, the training of the equilibrium´s capacity (a specific ability required for Dance Sport performance) increases the vestibular control of the body, as shown by the lower frequency shifts [51].

It has been shown that young ballet dancers reduced body sway, particularly during the second interval of the 30 s test, by reducing sway velocity (total and anterior posterior) and sway amplitude [47]. The shift toward a somatosensory control in adult dancers has been also confirmed by other studies [20,21,52].

Our results are in accordance with these previous studies. The correlation we found of spectral parameters with body height in children means that the older the subjects, the smaller and lower the frequency of oscillation, which translates to a better control of equilibrium [12,16,53].

The absence of correlations of spectral parameters in the Adult—Height analysis is different from the results of other studies [23] performed in a non-dancer’s population, while it agrees with the conclusions of other authors [24] regarding young adults. Based on these results, we can state that practice and high-level training in dancers improves balance to the point of counterbalancing the influence of height. In contrast with previous findings [11], we did not find any differences between males and females subjects for any of the sway parameters considered. These results are consistent with the hypothesis that dance training counteracts the gender differences in balance.

Since both children and adults are professional dancers in our groups, it can be presumed that the results are influenced by high-level practice and training as observed in other studies on ballroom dancers [51] and in other sports [52]. Furthermore, the variables we considered seem influenced by the stage of maturity of the nervous system [4,5,52]. For training purposes, we recommend training the somato-sensorial system to improve balance in children dancers, while exercises which challenge the vestibular system are recommended in adult dancers, who develop exceptional somato-sensory balance abilities during their growth and training history.

## 5. Conclusions

Our findings confirm that the following: (1) children rely mostly on the somatosensory system and adults on the vestibular system to maintain static monopodalic balance, (2) training in Dance Sport has a great effect in overcoming the limitations provided by anthropometry and by gender. The trainability theory states that each stage of development is sensitive to specific training, for a specific motor capability [54]. A recommendation for training, thus can be to use exercises with eyes open in children (less vestibular support) and with eyes open in adults. The limitations of our study are, as is the case for most of the previous research on the topic, that we measured balance in lab condition and non in real life conditions, and the limited number of subject studied, although due to the nature of the study (high level/high training ballroom dancers) recruitment was very difficult. Another limitation of our study is that we did not assess the Tanner stage of participants.

In conclusion, this study presents avenues for other questions and further potential research on the subject. It would be interesting to investigate the extent to which and the manner in which the balance capacity integrates with other elements of performance in ballroom dancers (i.e., resistance, body mobility, artistic capacities), and to identify the specific techniques that are most effective in training body balance. In addition, the influence of different heel heights, commonly worn in ballroom dance, should be investigated to assess the influence of heel height on balance.

## Figures and Tables

**Table 1:** Subjects’ data.

Adults	Age(Years)	Height (cm)	Weight (kg)	BMI	Dance Experience (Years)
Males (nr. 7)	26.5 ± 5	176.1 ± 9.2	66.3 ± 12	21.0 ± 1.4	10 ± 4
Females (nr. 8)	26.4 ± 8.5	166.8 ± 8.3	54.0 ± 10	19.9 ± 1.1	12 ± 3
Children					
Males (nr. 6)	10.6 ± 2.1	140.6 ± 10.5	35.1 ± 6.2	18.7 ± 3.2	5 ± 2
Females (nr. 7)	8.9 ± 2.3	135.9 ± 11	30.8 ± 4	16.6 ± 4	4 ± 2

Data are reported as Means and standard deviations.

**Table 2:** Frequency bands of the Tetrax system and the corresponding balance mechanism involved [37].

Frequency Band (Hz)	Balance System
F1 = 0.01–0.1	oculomotor vestibular otolythic
F2 = 0.1–0.25	vestibular
F3 = 0.25–0.35	vestibular
F4 = 0.35–0.50	vestibular
F5 = 0.50–0.75	somatosensory
F6 = 0.75–1.00	somatosensory
F7 = 1.00–3.00	central
F8 = 3.00 and above	central

**Table 3:** Correlation of body height in children with shift and spectral parameters.

Parameter	r	*p*
Shift range AP	−0.484	0.047
Equivalent Area	−0.484	0.047
AP oscillation frequency	−0.643	0.009
Max D power	−0.505	0.039

**Table 4:** Shift parameters and significant differences between Children and Adults.

Shift Parameter	Children		Adults		F	Sig.
	mean	sd	mean	sd		
Shift range (M-L) mm	61.3	43.6	46.7	11		
Shift range (A-P) (mm)	88.6	55.3	35.6	12.2	13.07	0.001
Total length (mm)	1815.5	836.9	1277.4	309	5.38	
Shift speed (mm/s)	59.3	27.1	41.6	10	5.38	
Equivalent area (mm^2^)	13,180.8	19,992.7	4357.59	1725.25		
Max M-L power (mm^2^)	5288.8	12,263.6	1632.4	1072.5		
Frequency to max M-L power (Hz)	0.08	0.03	0.04	0.02	11.03	0.003
Average M-L Power (mm^2^)	55.5	105.4	18.1	9		
Average M-L Frequency (Hz)	0.5	0.2	0.10	0.1		
Max A-P power (mm^2^)	3642	9494.7	308.7	135.9		
Frequency to max A-P power (Hz)	0.04	0.02	0.04	0.02		
Average A-P Power (mm^2^)	58.2	122.5	8.8	3.6		
Average A-P Frequency (Hz)	0.7	0.1	0.6	0.1		
Max D power (mm^2^)	1288.1	3020.4	1093.7	2839.6		
Frequency to max D power (Hz)	0.1	0.08	0.2	0.1		
Average D Power (mm^2^)	23.9	38.4	6.1	3.4		
Average D Frequency (Hz)	0.7	0.1	0.8	0.3		

Max M-L = middle-lateral oscillation, Max A-P = anterior-posterior oscillation, Max-D = oscillation with respect to its centerline. Alpha level: 0.025.

## Data Availability

Data available on request due to restrictions (privacy). The data presented in this study are available on request from the corresponding author. The data are not publicly available due to restriction imposed by University of Bologna on children’s data.
